# Exploring peculiarities and performance predictors of character strengths in individual and team sports

**DOI:** 10.1038/s41598-025-96230-0

**Published:** 2025-04-07

**Authors:** Nina Ramona Riedl, Petra Jansen, Stefanie Klatt

**Affiliations:** 1 Section Cognition in Team Sports, German Sport University Cologne, 50933 Cologne, Germany; 2 Department of Sports Science, University of Regensburg, 93053 Regensburg, Germany

**Keywords:** Positive psychology, Personality, Teamwork, Love of learning, Athlete, Human behaviour, Psychology

## Abstract

Research has indicated that character strengths are relevant to well-being and performance across various life domains; however, they have rarely been considered in the context of sports. The present study examined the potential of character strengths, meaning positive and personally fulfilling traits, in predicting (a) participation in individual/team sports and (b) competition levels among athletes in both individual and team sports. A sample of 683 adults (50.7% women; mean age = 27.9 years), including individual (*n* = 284) and team sports (*n* = 399) athletes engaged in different sports and competition levels, completed self-reports assessing their sporting backgrounds. Additionally, the VIA-120 (Values in Action Inventory of Strengths) was employed to measure the 24 character strengths in the VIA classification. The results reveal that (a) a set of character strengths significantly contributes to predicting individual/team sport participation and (b) adding character strengths to the prediction of performance levels leads to a small, albeit non-significant, increase in explained variances. These findings help guide future research on the mechanisms linking character strengths and sports participation and inform the design of personality development and other interventions in sports. While valuable insights are provided, character strengths research in sports should be expanded to include well-being-related outcomes and different methodologies.

## Introduction

In sports, the importance of strong character is widely discussed among athletes, coaches, and spectators and it is often regarded as the foundation for success within the realm of athletic competition. Personality research in sports has examined whether certain personality traits distinguish different categories of athletes. Frequently, the Five-Factor model of personality (FFM)^1^ served as the basis for this research. While team sport athletes are, on average, more extroverted and less conscientious than individual sport athletes, for instance, athletes competing at national or international levels were found to be more emotionally stable, agreeable, and conscientious in comparison to their counterparts competing at regional levels^2^. However, while some evidence points to the relevance of personality in the sports context, large research gaps still remain^2^.

As Riedl and Klatt^3^ suggested, research on the character strengths (CS)^4^ might help fill these gaps. CS are defined as positive traits or capacities which are personally fulfilling, valued across cultures, and linked to various positive outcomes for oneself and others^5^. A total of 24 CS are encompassed in the general classification of positive human traits^4^. Regarding its asset-building approach and emphasis on developing strengths and potential, research into CS in the context of sports bears similarities to research on positive (personality) development through sports. For instance, the character is considered one of the main indicators of thriving youths in the 5Cs model of positive youth development^6^. However, confirmatory factor analysis failed to support the 5Cs in a youth sports context^7^.

Furthermore, the concepts can be considered unique despite overlapping aspects of some CS with the Big Five traits^8^. McGrath et al.^9^ examined the incremental validity of CS over commonly measured personality facets (e.g., FFM^1^) in predicting various criterion measures and concluded that while the variables appear strongly related, the two models are not redundant. As CS are understood and shown to be changeable and trainable^4,10^, their value in sports may extend beyond the selection of athletes for certain teams/programs or the adaptation of intervention programs to athletes’ personalities. CS may also represent appropriate target variables for various sports-related interventions^3^.

The CS have been linked to crucial outcomes related to well-being and performance across various life domains^5,10^. Studies also demonstrate relations between CS and other positive performance-related outcomes. Among others, associations with satisfaction, engagement, positive behavior, and performance were found in the work^11^ and in the academic context^12,13^. The relations between CS and positive outcomes in physical activity or sports have only been investigated in a few studies: for instance, positive relationships between CS and subjective and physical well-being, were found in an adult German-speaking sample, with health-behaviors functioning as potential mechanisms^14^. Further, positive relationships between CS and physical fitness and activity were found among primary school children in Japan^15^. In both cases, the samples did not specifically consist of athletes. Another study conducted with elite and recreational youth athletes in Argentina showed that positive family functioning is related to the development of CS^16^, however, this study focused on the family context rather than sporting outcomes. Moreover, the latter two studies^15,16^ only investigated a selection of a few CS. Finally, a study by Tomé-Lourido et al.^17^ explored the effects of an 8-week CS intervention program in a Spanish professional soccer club and found improvements in athletes’ seasonal performance satisfaction and mood following the intervention.

Given the lack of initial research on CS in the sports context to date, the present study adopts an exploratory approach to investigating the role of CS in sports. The purpose of the study was twofold. Firstly, it aimed to explore if CS play a role in whether a person is more likely to participate in individual or team sports. More precisely, the objective was to determine the contributions of the CS in predicting team sport in contrast to individual sport participation. Note that for convenience, we classify athletes from integrated or segregated sport teams as team sport athletes and those involved in contrient sports as individual sport athletes, following the classical differentiation, as suggested by Evans et al.^18^. Secondly, the study aimed to explore the relevance of CS to athletic performance levels; this analysis was conducted separately for the team and individual sports athletes to identify differences between these groups. Based on previous personality research in sports^2^, we assumed that CS, in general, would contribute to predicting individual and team sport participation and athletic competition levels. However, we refrained from making specific predictions regarding which CS would be most relevant in this context.

## Method

### Participants

A total of 683 active athletes (337 men, 346 women; *M*_age_ = 27.87 years; *SD* = 10.42 years) participated in the study. The sample is comprised of individual (*n* = 284) and team (*n* = 399) athletes. All participants indicated that they regularly practiced their main sport. While some of them were recreational athletes who did not actively participate in competitions (*n* = 229), the majority (*n* = 454) were active competitors at various performance levels. All competition levels, from the lowest to the highest German leagues and international competitions, were represented in the sample. Participants indicated various main sports including soccer, handball, track and field, gymnastics, and several others.

### Measures

#### Demographic data and sporting background

Participants indicated their nationality, age, and gender (female, male, divers, no response). Since all participants identified as female (= 1) or male (= 2), gender was considered a dichotomous variable in all subsequent analyses. Furthermore, information on participants’ sporting backgrounds was collected, including their main sport, average weekly training duration, and competition level. Athletes were categorized as either individual (= 1) or team (= 2) sport participants. Moreover, athletes were classified into ten groups based on their current competitive levels according to the German sports league system (1 = no competition; 2 = lowest German/recreational league/Kreisebene; 3 = Bezirksebene; 4 = Landes-/Verbandsebene; 5 = Oberliga; 6 = Regionalliga; 7 = 3. Bundesliga; 8 = 2. Bundesliga; 9 = highest German league/national competitions/1. Bundesliga; 10 = international competitions).

#### Character strengths

The German version of the 120-item Values in Action Inventory of Strengths (VIA-IS; VIA Survey^4,19^) was used to assess the 24 CS. The VIA-120 measures these strengths, grouped under six virtues: Wisdom, which encompasses strengths related to knowledge acquisition and application, including creativity, curiosity, judgment, love of learning, and perspective; Courage, which included the strengths of bravery, perseverance, honesty, and zest; Humanity, reflecting interpersonal strengths such as love, kindness, and social intelligence; Justice, which refers to civic strengths like teamwork, fairness, and leadership; Temperance, including forgiveness, humility, prudence, and self-regulation; and Transcendence, which includes strengths that connect individuals to meaning and purpose, such as appreciation of beauty and excellence, gratitude, hope, humor, and spirituality.

The questionnaire consists of 120 items, with five items per scale, rated on a 5-point Likert scale ranging from 1 (= very much unlike me) to 5 (= very much like me). Each character strength score is calculated as the mean of its associated five items, with higher scores indicating stronger tendencies toward that strength. The questionnaire has an average completion time of approximately 15 to 20 min, serving as an abbreviated version of the original 240-item VIA-IS^4,19^. The German version of the VIA-120 is based on the English VIA-120 validation study by Littman-Ovadia^20^. The German VIA-120 was validated by Höfer et al.^21^, who concluded that its validity and reliability are comparable to those of the original VIA 240-item version.

### Procedure

The study was approved by the local ethics committee of the German Sport University Cologne, in Germany (no. 167/2021) and was conducted in accordance with the Declaration of Helsinki 1975. Participants were recruited online and through advertisements at the GSU Cologne, highlighting the requirement for participation of regular practice of one main sport. All participants provided informed consent before participating in the study. The survey was conducted online using the Questback EFS Survey© platform for data collection. Participants provided background information and filled in the VIA-120. Respondents did not receive any payment for their participation; however, they received feedback on their individual results.

### Data analysis

IBM SPSS Statistics (Version 29) was used for all statistical analyses. Data was analyzed at the specific CS level.

A hierarchal binary logistic regression was conducted to address the first research objective (i.e., determining the contributions of CS in predicting team/individual sport participation). To address the second research objective (i.e., determining contributions of CS in predicting competition levels of (a) individual and (b) team sport athletes), two hierarchical multiple regressions were performed. For these analyses, competition level was treated as a numerical variable, and therefore linear regressions were employed. In each regression, age and gender were entered in the first block, and the CS were added in the second. The chosen approach was derived from the methodology employed in prior research^22^. To test the robustness of the presented models, post hoc sensitivity analyses were conducted which are presented in the supplementary material.

## Results

Descriptive statistics for the sample are presented in Table 1.

**Table 1:** Descriptive data by team and individual sports participation.

	Individual (*n* = 284)	Team (*n* = 399)
Age in years (SD)	31.19 (13.60)	25.50 (6.40)
Gender		
Male	85 (29.9%)	252 (63.2%)
Female	199 (70.1%)	147 (36.8%)
Competitive levels		
1: No competitions	173 (60.9%)	55 (13.8%)
2: Kreisebene	29 (10.2%)	74 (18.5%)
3: Bezirksebene	0 (0%)	68 (17%)
4: Landes-/Verbandsebene	22 (7.7%)	65 (16.3%)
5: Oberliga	4 (1.4%)	60 (15%)
6: Regionalliga	9 (3.2%)	26 (6.5%)
7: 3. Bundesliga	3 (1.1%)	7 (1.8%)
8: 2. Bundesliga	3 (1.1%)	16 (4%)
9: 1. Bundesliga/national competitions	28 (9.9%)	20 (5%)
10: International competitions	13 (4.6%)	8 (2%)

*N* 683.

### Character strengths in individual and team sports

The data of all 683 participants was analyzed. The hierarchical binary logistic regression model was statistically significant at step one, χ^2^(2) = 127.11, *p* < 0.001, resulting in a small amount of explained variance, as shown by Nagelkerke’s R^2^ = 0.23. Adding the CS in the second block, led to a significant increase in explained variance of team sport participation, Δχ^2^(24) = 127.05, *p* < 0.001, ΔR^2^ = 0.19.

The overall model is statistically significant χ^2^(26) = 254.16, *p* < 0.001. A medium amount of variance, as shown by Nagelkerke’s R^2^ = 0.42, is explained by the overall model.

Of all variables entered into the regression model, seven contributed significantly in predicting team sport participation in the overall model, including the demographic variables age and gender and the CS of a*ppreciation of beauty and excellence*, *love of learning*, *teamwork*, *fairness*, and *humility*. All model coefficients and odds can be found in Table 2.

**Table 2:** Model coefficients of the hierarchical binary logistic regression on team sports membership.

Step	Predictor	*B*	*SE B*	Wald	*p*	OR	95% CI	
							LL	UL
1	Age	− 0.06	0.01	40.95	< 0.001*	0.94	0.92	0.96
	Gender	1.46	0.18	70.02	< 0.001*	4.32	3.07	6.09
2	Age	− 0.07	0.01	37.50	< 0.001*	0.93	0.91	0.95
	Gender	1.36	0.21	42.38	< 0.001*	3.90	2.59	5.87
	Appreciation	− 0.65	0.19	11.41	< 0.001*	0.52	0.36	0.76
	Bravery	0.03	0.21	0.02	0.891	1.03	0.69	1.54
	Creativity	0.15	0.19	0.60	0.441	1.16	0.80	1.68
	Curiosity	− 0.16	0.26	0.38	0.540	0.85	0.52	1.41
	Fairness	− 0.64	0.27	5.70	0.017*	0.53	0.31	0.89
	Forgiveness	0.03	0.20	0.02	0.879	1.03	0.70	1.51
	Gratitude	− 0.09	0.25	0.15	0.703	0.91	0.56	1.48
	Honesty	0.12	0.31	0.14	0.704	1.12	0.62	2.05
	Hope	0.29	0.24	1.42	0.233	1.33	0.83	2.13
	Humility	− 0.46	0.20	5.50	0.019*	0.63	0.43	0.93
	Humor	0.22	0.19	1.31	0.253	1.24	0.86	1.80
	Judgment	0.09	0.24	0.13	0.717	1.09	0.68	1.74
	Kindness	0.06	0.27	0.05	0.820	1.06	0.63	1.79
	Leadership	0.47	0.26	3.24	0.072	1.60	0.96	2.66
	Love	0.10	0.19	0.30	0.587	1.11	0.77	1.59
	Love of Learning	− 0.52	0.15	12.40	< 0.001*	0.60	0.45	0.80
	Perseverance	− 0.12	0.21	0.32	0.570	0.89	0.58	1.35
	Perspective	− 0.15	0.20	0.55	0.458	0.86	0.58	1.28
	Prudence	0.15	0.23	0.39	0.532	1.16	0.73	1.82
	Self-Regulation	− 0.30	0.16	3.28	0.070	0.74	0.54	1.02
	Social Intelligence	− 0.01	0.23	< 0.01	0.964	0.99	0.63	1.55
	Spirituality	0.06	0.13	0.19	0.663	1.06	0.83	1.35
	Teamwork	1.72	0.28	37.05	< 0.001*	5.60	3.22	9.75
	Zest	− 0.32	0.27	1.42	0.234	0.73	0.43	1.23

Degrees of freedom were 1 for all Wald statistics.

The overall model presented a good model fit, as indicated by the Hosmer–Lemeshow-Test, χ^2^(8) = 6.91, *p* > 0.05.

*N* 683, *SE B* Standard error of B, *OR* Odds Ratio, *CI* Confidence Interval.

*p < 0.05.

### Character strengths and competition level

#### Competition level in individual sports

Only the individual sport athletes’ (*n* = 284) data was analyzed. The hierarchical multiple linear regression model was statistically significant at step one, *F*(2, 281) = 8.79, *p* < 0.001, R^2^ = 0.06 (adjusted R^2^ = 0.05), accounting for a small amount of explained variance^23^. Adding the CS in the second block, led to a non-significant increase in explained variance of ΔR^2^ = 0.08, Δ*F*(24, 257) = 1.00, *p* = 0.474. The overall model was statistically significant *F*(26, 257) = 1.60, *p* = 0.038, R^2^ = 0.14 (adjusted R^2^ = 0.05), and accounted for 13.9% of the variance, representing a medium effect size (Cohen’s *f*^2^ = 0.16)^23^.

The analysis showed that besides age, the CS of *love of learning*, and *love* significantly contributed to predicting athletic competition level in the overall model. Note that robust standard errors (HC4 method) were used for the analysis. All model coefficients can be found in Table 3.

**Table 3:** Model coefficients of the hierarchical multiple linear regression on competition level in individual sports.

Step	Predictor	*B*	*SE*^a^* B*	*t*	*p*	95% CI	
						LL	UL
1	Age	− 0.05	0.01	− 5.14	< 0.001*	− 0.07	− 0.03
	Gender	0.39	0.41	0.96	0.339	− 0.41	1.20
2	Age	− 0.05	0.01	− 4.07	< 0.001*	− 0.08	− 0.03
	Gender	0.50	0.43	1.16	0.247	− 0.35	1.35
	Appreciation	− 0.22	0.37	− 0.58	0.564	− 0.95	0.52
	Bravery	0.27	0.33	0.82	0.413	− 0.38	0.92
	Creativity	0.22	0.34	0.65	0.516	− 0.45	0.90
	Curiosity	0.09	0.52	0.18	0.857	− 0.94	1.13
	Fairness	0.18	0.45	0.39	0.698	− 0.72	1.07
	Forgiveness	0.23	0.33	0.69	0.489	− 0.42	0.88
	Gratitude	0.24	0.44	0.54	0.588	− 0.62	1.10
	Honesty	− 0.42	0.63	− 0.66	0.508	− 1.67	0.83
	Hope	− 0.43	0.47	− 0.92	0.361	− 1.35	0.49
	Humility	0.60	0.38	1.61	0.109	− 0.14	1.34
	Humor	− 0.40	0.36	− 1.10	0.271	− 1.12	0.32
	Judgment	− 0.36	0.43	− 0.86	0.393	− 1.20	0.47
	Kindness	− 0.68	0.47	− 1.45	0.147	− 1.61	0.24
	Leadership	− 0.42	0.46	− 0.91	0.364	− 1.32	0.49
	Love	0.65	0.33	1.99	0.048*	0.01	1.29
	Love of Learning	0.57	0.28	2.00	0.046*	0.01	1.12
	Perseverance	0.61	0.44	1.40	0.162	− 0.25	1.47
	Perspective	− 0.33	0.40	− 0.81	0.417	− 1.12	0.47
	Prudence	0.05	0.39	0.13	0.896	− 0.72	0.82
	Self-Regulation	− 0.55	0.29	− 1.91	0.058	− 1.12	0.02
	Social Intelligence	− 0.26	0.40	− 0.64	0.522	− 1.05	0.53
	Spirituality	− 0.08	0.28	− 0.29	0.771	− 0.62	0.46
	Teamwork	− 0.11	0.39	− 0.29	0.773	− 0.87	0.65
	Zest	0.27	0.48	0.56	0.576	− 0.67	1.21

*n* 284, *SE* Standard error of B, *CI* Confidence interval.

^a^robust standard errors using the HC4-method.

*p < 0.05.

#### Competition level in team sports

Only the team sport athletes’ (*n* = 399) data was analyzed. The hierarchical multiple linear regression model was statistically significant at step one, *F*(2, 396) = 9.43, *p* < 0.001, R^2^ = 0.05 (adjusted R^2^ = 0.04), accounting for a small amount of explained variance^23^. Adding the CS in the second block led to a non-significant increase in explained variance of ΔR^2^ = 0.08, Δ*F*(24, 372) = 1.32, *p* = 0.143. The overall model was statistically significant *F*(26, 372) = 1.96, *p* = 0.004, R^2^ = 0.12 (adjusted R^2^ = 0.06), and accounted for 12.1% of the variance, representing a small effect size (Cohen’s *f*^2^ = 0.14)^23^.

The analysis showed that besides age, the CS of *teamwork* significantly contributed to predicting competition level in team sports in the overall model. Note that robust standard errors (HC4 method) were used for the analysis. All model coefficients can be found in Table 4.

**Table 4:** Model coefficients of the hierarchical multiple linear regression on competition level in team sports.

Step	Predictor	*B*	*SE*^a^* B*	*t*	*p*	95% CI	
						LL	UL
1	Age	− 0.06	0.02	− 4.30	< 0.001*	− 0.09	− 0.03
	Gender	− 0.53	0.25	− 2.17	0.031*	− 1.02	− 0.05
2	Age	− 0.08	0.02	− 4.93	< 0.001*	− 0.11	− 0.05
	Gender	− 0.56	0.31	− 1.85	0.065	− 1.16	0.04
	Appreciation	− 0.16	0.23	− 0.69	0.492	− 0.60	0.29
	Bravery	0.07	0.29	0.24	0.810	− 0.50	0.63
	Creativity	0.26	0.22	1.19	0.236	− 0.17	0.68
	Curiosity	− 0.03	0.32	− 0.08	0.936	− 0.65	0.60
	Fairness	− 0.20	0.32	− 0.60	0.550	− 0.84	0.45
	Forgiveness	− 0.28	0.25	− 1.09	0.276	− 0.78	0.22
	Gratitude	− 0.34	0.32	− 1.03	0.302	− 0.97	0.30
	Honesty	− 0.04	0.37	− 0.10	0.923	− 0.75	0.68
	Hope	− 0.07	0.30	− 0.23	0.815	− 0.67	0.52
	Humility	0.38	0.21	1.80	0.073	− 0.04	0.80
	Humor	− 0.11	0.24	− 0.48	0.634	− 0.58	0.36
	Judgment	0.25	0.32	0.78	0.439	− 0.38	0.88
	Kindness	− 0.36	0.31	− 1.16	0.245	− 0.97	0.25
	Leadership	0.24	0.31	0.78	0.434	− 0.37	0.86
	Love	0.03	0.22	0.13	0.894	− 0.41	0.47
	Love of Learning	0.02	0.18	0.10	0.922	− 0.33	0.36
	Perseverance	0.28	0.25	1.13	0.261	− 0.21	0.77
	Perspective	− 0.23	0.27	− 0.86	0.390	− 0.75	0.29
	Prudence	− 0.55	0.30	− 1.88	0.062	− 1.14	0.03
	Self-Regulation	0.04	0.20	0.22	0.823	− 0.35	0.43
	Social Intelligence	0.07	0.29	0.26	0.795	− 0.49	0.63
	Spirituality	0.05	0.15	0.31	0.756	− 0.24	0.33
	Teamwork	1.18	0.35	3.38	< 0.001*	0.49	1.86
	Zest	0.23	0.33	0.70	0.486	− 0.42	0.88

*n* 399, *SE* Standard error of B, *CI* Confidence interval.

^a^robust standard errors using the HC4-method.

*p < 0.05.

## Discussion

The present study extends earlier research on CS by examining their role in the specific context of sports. The present results indicate that a set of CS and demographic variables significantly predict team/individual sports participation. First, higher age is associated with individual sports participation. This finding corresponds to existing data showing that middle age is associated with participation in individual, unorganized physical activities (e.g., running, fitness), while, for example, soccer participation is associated with younger age^24^. Further, male gender increases the likelihood of team sports participation. This aligns with data on sports participation in Germany, indicating that men prefer team sports with a ball (e.g., soccer). In contrast, women tend to favor sports that focus on coordinative aspects (e.g., Pilates/yoga)^25^.

The CS of *teamwork* is associated with an increased likelihood of team sport participation. *Teamwork* is defined as being a loyal group member, contributing one’s share, and generally working well as a team member^4^*.* Reasonably, a person with this strength might choose to participate in a sport that requires exactly those skills. Previous research has shown that social and affiliation motives (i.e., doing something in a group, making or meeting friends/acquaintances) are particularly relevant motives for sports participation among team sport athletes^26^.

Conversely, individual sports athletes are not dependent on the cooperative actions of other athletes to perform their tasks^27^. Higher levels of *love of learning*, *appreciation of beauty and excellence*, *fairness*, and *humility* were found to be associated with an increased likelihood of individual sport participation. The CS of *love of learning* is about mastering new skills or bodies of knowledge^4^. Individual sports tend to require more closed skills (i.e., greater focus on the movements), whereas team sports oftentimes require the ability to react to changes in the environment (e.g., teammates’ and opponents’ actions) and therefore involve more open skills^28^. Thus, more *love of learning* may be required to train and optimize certain movement skills/patterns that play a more decisive role in individual sports (e.g., preparation for a perfectly rehearsed choreography in gymnastics). This explanation would also align with previous research, showing skill development to be a significantly more pronounced factor in the sports participation motivation of individual compared to team sport athletes^29^.

The CS of *appreciation of beauty and excellence* entails appreciating beauty, excellence, and/or skilled performance in various life domains^4^. Appreciation of physical beauty^5^ may be particularly relevant to explaining the importance of the strength of *appreciation* in individual sports. For instance, it may lead athletes to participate in lean sports. Lean sports are those in which leanness and/or low bodyweight play a role in athletes’ performances^30^. Leanness is often associated with individual sports like distance running, weightlifting, and gymnastics. In contrast, sports defined as non-lean (where leanness does not lead to advantages and participants, have not judged aesthetically) are mainly associated with team sports like soccer or basketball^31^.

The finding that *fairness*, which entails giving everyone a fair chance, treating everyone the same, and not letting one’s personal feelings bias decisions about others^4^, is a predictor of individual as opposed to team sport participation is somewhat surprising. Fairness, respect, and tolerance are considered universally valid ethical and moral behaviors and social skills that are among the Olympic values and goals of the Olympic Education, regardless of individual or team sport. One explanation for this finding could be that individual sports often rely on objective criteria (e.g., time, distance, points), while team sports frequently encompass external factors and subjective judgments, such as coaching decisions and referee calls. The literature provides ample evidence of influences and biases on the judgments of these stakeholders in sports^32^. Moreover, individual performances are more easily discernible and decisive in individual sports. In team sports, distinguishing individual performances is challenging due to behavioral interdependence^27^. Consequently, individual sports athletes may perceive their performance as more directly linked to their efforts and less affected by subjective decisions, perceive opportunities and conditions for success to be more equal than in team sports, and thus choose to participate in individual sports.

Finally, as individual sports athletes have a more direct impact on their performances, these sports demand high personal accountability. The CS of *humility* involves not viewing oneself as more special than others and letting one’s achievements speak for themselves^4^. *Humility* helps in recognizing personal strengths and weaknesses, taking responsibility for mistakes, and being open to learning and self-improvement^33^. Acknowledging one’s limitations and the need for growth are crucial factors for personal development, particularly in individual sports.

Understanding personality can enhance interpersonal relationships and inform training and intervention strategies for athletes^2^. For instance, the underuse and overuse of strengths are linked to negative outcomes like depression and anxiety^34^. Thus, it is worth examining if strengths overuse or underuse relates to negative sport-related behaviors such as overtraining or unhealthy weight management. Understanding CS in sports could help identify at-risk athletes and develop prevention strategies for such behaviors.

Finally, the present findings may serve as the basis for exploring the causes of group-based differences in sports. Longitudinal studies could investigate whether participation in various sports affects the development of different CS, offering insights into positive youth development and the character-building role of sports. Future studies might also assess whether providing athletes with opportunities to use their CS in relevant contexts enhances satisfaction, well-being, and decreases drop-out rates.

The second focus of the current study was to investigate the links between CS and athletic performance, as approximated by self-reported athletic competition level. Athletes’ age predicted lower competition levels in individual and team sports, accounting for a small amount of explained variance. On average, elite athletes retire around the age of 34 after competing at the highest levels for no more than 5–10 years^35^. Moreover, age is positively related to the importance of health-related motives for sports participation and negatively related to competition-related motives^26^. Therefore, it appears that with increasing age, athletes compete at lower performance levels or drop out of competitive sports.

The CS did not significantly predict competition levels in team sports or individual sports, albeit small increases in explained variances were noted, and the overall models were significant. In team sports, only *teamwork* significantly predicted higher competition levels, highlighting its importance in completing tasks and outperforming other teams^27^. In individual sports, the overall model was significant, with *love of learning* and *love* significantly predicting higher competition levels. Both strengths are linked to autonomy and effective coping with stress^5^, which are important for the motivation, resilience, and performance of athletes. However, the non-significant increases in explained variances suggest that the relevance of these CS to sports performance should be interpreted with caution. These findings should be understood in the context of the exploratory nature of this study and the limited predictors included in the models. The emergence of only a few, certain CS as significant predictors emphasize the complexity of athletic performance, which likely stems from interactions among numerous unmeasured factors. Future research should aim to address these limitations by utilizing larger sample sizes or adopting alternative methodologies, such as qualitative approaches, to deepen the understanding of the relationships between CS and sports performance. Given the exploratory nature of this study, replication is strongly recommended. Adjusted R^2^ values were calculated for both multiple regression models to account for the large number of predictors. Although the adjusted R^2^ values of both models were low, it is important to note that low R^2^ values are not inherently negative, particularly in exploratory research and studies involving complex social phenomena. This outcome reflects the addition of the CS, of which only two and one, respectively, were identified as significant predictors.

The interpretation of the present findings is also limited by the cross-sectional nature of the study. In certain cases, it appears plausible that CS may influence athletic performance or the choice of participating in a team or an individual sport. Conversely, it is conceivable that these strengths are influenced/developed by the specific environments associated with high-performance or individual/team sports^4^. Longitudinal studies are needed to test such hypotheses. Moreover, it cannot be ruled out that other confounding variables explain the relationship between CS and sport participation and competition level. Future studies should include additional factors known to be related to sports participation (e.g., sports participation motives^26^ or the Big Five traits^1^) and assess the incremental predictive ability of CS in such models.

As no restrictions regarding the participants’ main sport existed, the sample included a wide range of different types of sports*.* While this can be seen as increasing the generalizability of the results, it may also mask important differences between different types of sports, which were subsumed under the two broad categories of individual and team sports. Future studies might, therefore, take sport-specific approaches or focus on other narrower categories of sports (e.g., martial arts, acrobatic sports, sports games).

Future studies might also analyze the composition of sports teams regarding their CS profiles and examine how the (dis)similarity of team members’ profiles affects outcomes like team cohesion, satisfaction, or success. Previous studies have explored the effects of team members’ CS and team roles on team outcomes in work-related settings and found that teams with higher levels of certain strengths (i.e., *teamwork*, *fairness*, *prudence*) report higher levels of positive team outcomes^36^. However, in the sports context, the relationships between a team’s success and its members’ personalities still require urgent empirical attention, as pointed out by Allen et al.^2^.

Some limitations concerning the measurement tools used should be considered. The present study is based on self-report measures only, which are prone to different sources of inaccuracy, like social desirability. Of the CS only *prudence* and *spirituality* were shown to correlate significantly with social desirability and anonymous administration and the use of computerized tests can help reduce these biases^37^, both of which are accounted for in the present study.

The present study contributes to the literature on personality and CS research in the context of sports. First insights are provided into the relevance of certain CS for individual and team sports, and the foundation for future research is laid. The CS significantly contributed to the prediction of team versus individual sport participation. They did not significantly contribute to predicting competition levels in team and individual sports, albeit small contributions to explained variances were found, and overall models were significant. Nevertheless, future studies should corroborate the present findings and continue exploring the role of CS in sports regarding different subgroups of athletes and other positive well-being and performance-related outcomes. As CS are understood and shown to be changeable and trainable^4,10^, future research might determine their potential value as target variables for personality development and various other interventions in the sports context.

## Supplementary Information

Supplementary Information.

## Data Availability

Data are available in a public, open-access repository at https://osf.io/rh869/
