# Supplementary material for: Exploring peculiarities and performance predictors of character strengths in individual and team sports
Source: Sci Rep. 2025 Apr 7;15:11833. doi: 10.1038/s41598-025-96230-0 (PMC11977205; doi:10.1038/s41598-025-96230-0)
Supplement: Supplementary file 1 — Supplementary Information. [file 41598_2025_96230_MOESM1_ESM.docx]

**Supplementary Material**

**Methods**

**Measures**

***Character Strengths***

In the present study, McDonald’s omega was calculated to assess the internal consistencies of the VIA’s subscales, with values ranging between ω = .85 (*spirituality*) and ω = .53 (*leadership*).

**Results**

**Power Analysis**

With a small effect size of *f* ^2^ = 0.075, an alpha-level of α = .05, a power of 1-ß = 0.95, and 26 possible predictors (age, gender, and the 24 character strengths) for the dependent variable, a power analysis for the linear regression resulted in N = 470 (Faul et al., 2007).

**Descriptive Data**

The data of the 683 athletes showed that the character strength with the highest mean across the total sample was *honesty* (*M* = 4.28, *SD* = 0.42), followed by *kindness* and *fairness* (*M* > 4.0). The lowest mean across the total sample was found for *spirituality* (*M* = 2.42, *SD* = 0.92).

***Individual Sport Athletes***

Among the 284 (85 men, 199 women; *M_age_* = 31.19 years; *SD* = 13.60 years) individual sport athletes in this sample, the same pattern was found, and the character strength with the highest mean was *honesty* (*M* = 4.25, *SD* = 0.42), followed by *kindness* and *fairness* (*M* > 4.0). Furthermore, among the top five means were *judgement* and *social intelligence* (*M* > 3.9). The lowest mean was found for *spirituality* (*M* = 2.38, *SD* = 0.92). Descriptive values of the individual sport athlete sample can be found in Table 1.

***Team Sport Athletes***

The highest mean of the character strengths in the sample of the 399 (252 men, 147 women; *M_age_* = 25.50 years; *SD* = 6.40 years) team sport athletes was also *honesty* (*M* = 4.30, *SD* = 0.41), followed by *kindness* and *fairness* (*M* > 4.0). *Humor* and *social intelligence* (M > 3.9) were further among the top five means, while again, the lowest mean was found for *spirituality* (*M* = 2.45, *SD* = 0.92). Descriptive values of the team sport athlete sample can be found in the table below.

**Character Strengths in Individual and Team Sports**

A hierarchal binary logistic regression was performed to determine the contributions of the character strengths in predicting team sport membership (as opposed to individual sport membership). The assumptions of the binary logistic regression were met. Correlations between predictor variables were low (*r* < .46), indicating that multicollinearity was not a confounding factor in the analysis.

***Sensitivity Analysis***

The regression was repeated omitting all insignificant variables detected in the first regression. The binary logistic regression model, including the variables age, gender, appreciation of beauty and excellence, love of learning, teamwork, fairness, and humility was statistically significant, χ²(7) = 239.80, *p* < .001, resulting in a small amount of explained variance, as shown by Nagelkerke’s R² = .399. A good model fit was indicated by the Hosmer-Lemeshow-Test, χ²(8) = 14.16, *p* > .05. All model coefficients and odds can be found in Table S2.

Of the variables entered into the regression model, age, gender, appreciation of beauty and excellence, love of learning, and teamwork contributed significantly to predicting team sport participation. Thus, the regression was repeated, again omitting the insignificant predictors (i.e., fairness and humility). This model was statistically significant, χ²(5) = 226.76, *p* < .001, resulting in a small amount of explained variance, as shown by Nagelkerke’s R² = .38. A good model fit was indicated by the Hosmer-Lemeshow-Test, χ²(8) = 14.85, *p* > .05. All entered variables were significant predictors of team sport participation. All model coefficients and odds can be found in Table S2.

**Character Strengths and Competition Level**

***Competition Level in Individual Sports***

A hierarchical multiple linear regression was performed to determine the contributions of the character strengths in predicting the level of competition of individual sport athletes. The assumptions of the multiple linear regression model were examined. Tests to see if the data met the assumption of collinearity indicated that multicollinearity was not a concern (*r* < .70; VIF < 2.86). Although the White-Test for heteroscedasticity was not significant, χ²(283) = 284.00, *p* = .472, the visual analysis of the scatterplot of standardized residuals pointed to a possible violation of the assumption of homogeneity of variance. Therefore, the HC4 method (heteroscedasticity-consistent standard error estimator) was applied, and robust standard errors were used (Hayes & Cai, 2007).

***Sensitivity Analysis***

The regression was repeated omitting all insignificant variables detected in the first regression. The multiple linear regression model, including only the variables of age, and love was statistically significant, F(3, 280) = 6.58, *p* < .001, R² = .066 (adjusted R² = .056), accounting for a small amount of explained variance, representing a small effect size (Cohen’s *f*² = .07). All model coefficients can be found in Table S3. In this model, only age emerged as a significant predictor of competition level in individual sports. Thus, a linear regression was performed omitting the insignificant predictors and including only age. The simple linear regression was statistically significant, F(1, 282) = 16.52, *p* < .001, R² = .055 (adjusted R² = .052), accounting for a small amount of explained variance, representing a small effect size (Cohen’s *f*² = .06). All model coefficients can be found in Table S3.

***Competition Level in Team Sports***

A hierarchical multiple linear regression was used to determine the contributions of the character strengths in predicting the level of competition of team sport athletes. The assumptions of the multiple linear regression model were examined. Tests to see if the data met the assumption of collinearity indicated that multicollinearity was not a concern (*r* < .70; VIF < 2.47). Again, although the White-Test for heteroscedasticity was not significant, χ²(376) = 370.18, *p* = .575, the visual analysis of the scatterplot of standardized residuals pointed to a possible violation of the assumption of homogeneity of variance. Therefore, the HC4 method (heteroscedasticity-consistent standard error estimator) was applied, and robust standard errors were used (Hayes & Cai, 2007).

***Sensitivity Analysis***

The regression was repeated omitting all insignificant variables detected in the first regression. The multiple linear regression model, including only the variables of age and teamwork was statistically significant, F(2, 396) = 13.03, *p* < .001, R² = .062 (adjusted R² = .057), accounting for a small amount of explained variance, representing a small effect size (Cohen’s f² = .07). Both variables contributed significantly to predicting competition level in team sports. All model coefficients can be found in Table S4.

**Table S1 Supplementary Material**

*The 24 VIA-Character Strengths of Individual and Team Sport Athletes*

| VIA Character Strengths |  | Mean | *SD* | Min | Max | Skewness | Kurtosis |
| --- | --- | --- | --- | --- | --- | --- | --- |
|  |  | I \| T | I \| T | I \| T | I \| T | I \| T | I \| T |
| Appreciation of B & E |  | 3.56 \| 3.34 | 0.61 \| 0.62 | 1.8 \| 1.6 | 5.0 \| 5.0 | -0.14 \| 0.03 | -0.16 \| -0.04 |
| Bravery |  | 3.55 \| 3.59 | 0.63 \| 0.56 | 2.0 \| 2.2 | 5.0 \| 5.0 | -0.08 \| -0.22 | -0.20 \| -0.19 |
| Creativity |  | 3.41 \| 3.40 | 0.71 \| 0.62 | 1.6 \| 1.6 | 5.0 \| 5.0 | 0.06 \| -0.06 | -0.31 \| -0.09 |
| Curiosity |  | 3.86 \| 3.78 | 0.52 \| 0.54 | 2.0 \| 2.0 | 5.0 \| 5.0 | -0.12 \| -0.36 | -0.18 \| -0.07 |
| Fairness |  | 4.08 \| 4.10 | 0.52 \| 0.50 | 1.8 \| 2.2 | 5.0 \| 5.0 | -0.50 \| -0.54 | 0.67 \| 0.46 |
| Forgiveness |  | 3.64 \| 3.63 | 0.60 \| 0.56 | 1.8 \| 1.6 | 5.0 \| 5.0 | -0.12 \| -0.17 | 0.07 \| 0.13 |
| Gratitude |  | 3.64 \| 3.64 | 0.66 \| 0.57 | 1.6 \| 1.6 | 5.0 \| 5.0 | -0.09 \| -0.06 | -0.04 \| 0.00 |
| Honesty |  | 4.25 \| 4.30 | 0.42 \| 0.41 | 2.8 \| 2.8 | 5.0 \| 5.0 | -0.30 \| -0.63 | 0.20 \| 0.54 |
| Hope |  | 3.65 \| 3.71 | 0.66 \| 0.63 | 1.6 \| 1.4 | 5.0 \| 5.0 | -0.49 \| -0.81 | 0.23 \| 0.75 |
| Humility |  | 3.36 \| 3.32 | 0.58 \| 0.61 | 1.6 \| 1.6 | 4.8 \| 5.0 | 0.03 \| -0.34 | 0.12 \| -0.13 |
| Humor |  | 3.79 \| 3.99 | 0.67 \| 0.62 | 1.6 \| 2.0 | 5.0 \| 5.0 | -0.62 \| -0.48 | 0.25 \| -0.01 |
| Judgement |  | 3.94 \| 3.92 | 0.56 \| 0.53 | 2.0 \| 2.0 | 5.0 \| 5.0 | -0.45 \| -0.48 | 0.23 \| 0.25 |
| Kindness |  | 4.19 \| 4.25 | 0.50 \| 0.49 | 2.2 \| 2.4 | 5.0 \| 5.0 | -0.51 \| -0.53 | 0.65 \| 0.22 |
| Leadership |  | 3.72 \| 3.83 | 0.49 \| 0.46 | 2.2 \| 2.4 | 5.0 \| 5.0 | -0.22 \| -0.16 | 0.09 \| 0.12 |
| Love |  | 3.87 \| 3.02 | 0.71 \| 0.75 | 1.2 \| 1.4 | 5.0 \| 5.0 | -0.83 \| 0.26 | 1.02 \| -0.45 |
| Love of Learning |  | 3.47 \| 3.97 | 0.77 \| 0.64 | 1.4 \| 1.8 | 5.0 \| 5.0 | -0.14 \| -0.48 | -0.60 \| -0.17 |
| Perseverance |  | 3.83 \| 3.83 | 0.64 \| 0.60 | 1.8 \| 1.8 | 5.0 \| 5.0 | -0.39 \| -0.57 | 0.08 \| 0.37 |
| Perspective |  | 3.51 \| 3.57 | 0.59 \| 0.57 | 2.0 \| 2.0 | 5.0 \| 5.0 | 0.03 \| -0.11 | -0.07 \| -0.14 |
| Prudence |  | 3.50 \| 3.55 | 0.62 \| 0.59 | 1.6 \| 1.8 | 5.0 \| 5.0 | -0.19 \| -0.20 | -0.18 \| 0.02 |
| Self-Regulation |  | 3.32 \| 3.22 | 0.75 \| 0.71 | 1.2 \| 1.2 | 5.0 \| 5.0 | -0.11 \| -0.14 | -0.16 \| -0.23 |
| Social Intelligence |  | 3.91 \| 3.99 | 0.56 \| 0.54 | 2.2 \| 1.8 | 5.0 \| 5.0 | -0.43 \| -0.65 | -0.03 \| 0.55 |
| Spirituality |  | 2.38 \| 2.44 | 0.92 \| 0.92 | 1.0 \| 1.0 | 5.0 \| 5.0 | 0.70 \| 0.60 | 0.11 \| -0.15 |
| Teamwork |  | 3.68 \| 3.96 | 0.53 \| 0.47 | 2.0 \| 2.4 | 5.0 \| 5.0 | -0.22 \| -0.32 | 0.71 \| 0.44 |
| Zest |  | 3.65 \| 3.65 | 0.62 \| 0.57 | 1.6 \| 1.8 | 5.0 \| 5.0 | -0.33 \| -0.44 | 0.16 \| 0.14 |

*Note.* I: individual sport athletes (N = 284); T: team sport athletes (N = 399); SD: Standard deviation; Appreciation of B & E: Appreciation of Beauty and Excellence.

**Table S2**

*Model Coefficients of the Reduced Binary Logistic Regressions on Team Sports Membership*

| Model | Predictor | *B* | | *SE B* | Wald | *p* | *OR* | 95% CI | | |
| --- | --- | --- | --- | --- | --- | --- | --- | --- | --- | --- |
|  |  |  |  |  |  |  |  | LL | UL | |
| 1 |  |  |  |  |  |  |  |  | |  |
|  | Age |  | -0.07 | .01 | 35.92 | <.001* | 0.94 | 0.92 | | 0.96 |
|  | Gender |  | 1.43 | .19 | 55.34 | <.001* | 4.20 | 2.88 | | 6.12 |
|  | Appreciation |  | -0.56 | .17 | 11.40 | <.001* | 0.57 | 0.41 | | 0.79 |
|  | Love of Learning |  | -0.56 | .13 | 19.07 | <.001* | 0.57 | 0.45 | | 0.74 |
|  | Teamwork |  | 1.90 | .26 | 55.22 | <.001* | 6.70 | 4.06 | | 11.07 |
|  | Fairness |  | -0.46 | .23 | 4.06 | .044 | 0.63 | 0.40 | | 0.99 |
|  | Humility |  | -0.47 | .17 | 7.31 | .007 | 0.63 | 0.45 | | 0.88 |
| 2 |  |  |  |  |  |  |  |  | |  |
|  | Age |  | -0.06 | .01 | 34.06 | <.001* | 0.94 | 0.92 | | 0.96 |
|  | Gender |  | 1.40 | .19 | 54.70 | <.001* | 4.06 | 2.80 | | 5.88 |
|  | Appreciation |  | -0.67 | .16 | 17.14 | <.001* | 0.51 | 0.37 | | 0.70 |
|  | Love of Learning |  | -0.58 | .13 | 21.23 | <.001* | 0.56 | 0.44 | | 0.72 |
|  | Teamwork |  | 1.47 | .21 | 49.78 | <.001* | 4.33 | 2.88 | | 6.51 |

*Note.* N = 683, SE B = Standard error of B, OR = Odds Ratio, CI = Confidence Interval.

Degrees of freedom were 1 for all Wald statistics. **p* < .05.

**Table S3**

*Model Coefficients of the Reduced Multiple Linear Regression on Competition Level in Individual Sports*

| Model | Predictor |  | *B* | *SE^a^ B* | *t* | *p* | 95% CI | |
| --- | --- | --- | --- | --- | --- | --- | --- | --- |
|  |  |  |  |  |  |  | LL | UL |
| 1 |  |  |  |  |  |  |  |  |
|  | Age |  | -0.05 | .01 | -4.84 | <.001* | -0.07 | -0.03 |
|  | Love of Learning |  | 0.37 | .24 | 1.55 | .122 | -0.10 | 0.83 |
|  | Love |  | 0.23 | .22 | 1.06 | .291 | -0.20 | 0.66 |
| 2 |  |  |  |  |  |  |  |  |
|  | Age |  | -0.05 | .01 | -4.99 | <.001* | -0.07 | -0.03 |

*Note. n* = 284, SE = Standard error of B, CI = Confidence Interval.

a. robust standard errors using the HC4-method. **p* < .05

**Table S4**

*Model Coefficients of the Reduced Multiple Linear Regression on Competition Level in Team Sports*

| Model | Predictor |  | *B* | *SE^a^ B* | *t* | *p* | 95% CI | |
| --- | --- | --- | --- | --- | --- | --- | --- | --- |
|  |  |  |  |  |  |  | LL | UL |
| 1 |  |  |  |  |  |  |  |  |
|  | Age |  | -0.07 | .01 | -5.02 | <.001* | -0.10 | -0.04 |
|  | Teamwork |  | 0.83 | .25 | 3.38 | <.001* | 0.35 | 1.31 |

*Note.* *n* = 399, SE = Standard error of B, CI = Confidence Interval.

a. robust standard errors using the HC4-method. **p* < .05
